# Electrical Storm in Patients with Implantable Cardioverter-defibrillators: A Practical Overview

**DOI:** 10.19102/icrm.2017.081002

**Published:** 2017-10-15

**Authors:** Daniele Muser, Jackson J. Liang, Pasquale Santangeli

**Affiliations:** ^1^Electrophysiology Section, Cardiovascular Division, Hospital of the University of Pennsylvania, Philadelphia, PA

**Keywords:** Antiarrhythmic drugs, catheter ablation, electrical storm, mechanical hemodynamic support, ventricular tachycardia

## Abstract

Electrical storm (ES) is an increasingly common medical emergency characterized by clustered episodes of sustained ventricular arrhythmias (VAs) that lead to repeated appropriate implantable cardioverter-defibrillator (ICD) therapies. A diagnosis of ES can be made with the occurrence of three or more sustained episodes of VAs, or of three or more appropriate ICD therapies within 24 hours in patients with implanted devices. ES is associated with poor outcomes in patients with structural heart disease, particularly those with severe left ventricular dysfunction. In large clinical trials involving patients with ICDs for primary and secondary prevention, ES appears to be a predictor of cardiac death, with notably higher rates of mortality soon after the event. ES management is challenging and requires special medical attention with accurate patient risk stratification and a multidisciplinary approach that includes the use of pharmacologic therapies such as antiarrhythmic drugs (AADs) and interventional approaches like catheter ablation, surgical ablation, or sympathetic neuromodulation. Initial management involves determining and addressing the underlying ischemia, any electrolyte imbalances, and/or other causative factors. Hemodynamic support needs to be considered in high-risk patients with unstable VAs or those with severe comorbidities such as low left ventricular ejection fraction, advanced New York Heart Association class, and/or chronic pulmonary disease. Following the acute phase of ES, treatment should shift towards maximizing therapeutic efforts to address heart failure, performing revascularization, and preventing subsequent VAs. In the present manuscript, we offer an overview of the most relevant clinical aspects of ES with regard to novel therapeutic strategies.

## Introduction

Electrical storm (ES) is a life-threatening condition characterized by recurrent ventricular arrhythmias (VAs) requiring urgent medical care. Its current definition implies a condition in which three or more distinct episodes of sustained ventricular tachycardia/fibrillation (VT/VF) occur within 24 hours or the presence of continuous VT for at least 12 hours. In patients with implantable cardioverter-defibrillators (ICDs), ES is defined by three or more appropriate device interventions (each separated by at least five minutes), either with antitachycardia pacing (ATP) or ICD shock.^1^ Electrical storm can occur in several clinical scenarios such as in the acute phase of myocardial infarction, in the presence of structural heart disease and low left ventricular ejection fraction (LVEF), and in patients with inherited arrhythmic syndromes and structurally normal hearts (ie, in those with Brugada syndrome and catecholaminergic polymorphic VT).^2^ Successful ES management can be challenging and generally requires a tailored approach based upon an evaluation of the patient’s unique underlying heart disease and the severity of their clinical presentation. A multidisciplinary strategy comprised of correcting triggering factors, ICD programming, antiarrhythmic drug therapy (AAD), and invasive approaches (eg, catheter ablation (CA) and sympathetic denervation) is usually required to effectively suppress VAs and prevent recurrences. In this review, we summarize the most relevant clinical aspects of ES to date, as well as its therapeutic strategies, with special consideration given to interventional procedures.

## Incidence and predictors

The true incidence of ES in ICD carriers is difficult to estimate due to its heterogeneous definition (especially in older studies) and its relation to the underlying heart disease and reasons for ICD implantation. Using the current definition of the occurrence of at least three appropriate ICD interventions within a 24-hour period, the incidence of ES has been reported to be from 10% to 28% within the first three years after ICD implantation for secondary prevention.^3–5^ The incidence of ES in primary prevention ICD carriers appears to be lower, with 4% of patients developing ES over an average of 21 months in the Multicenter Automatic Defibrillator Implantation Trial II (MADIT-II) substudy that included 719 patients.^6^

Beyond acute arrhythmic triggers including ongoing ischemia, electrolyte imbalances, and sepsis, which must be ruled out in the initial evaluation of patients who present with ES, the clinical predictors of ES that can be used to identify high-risk patients requiring intensive monitoring are poorly understood. In a meta-analysis that included 5,912 patients from 13 studies, secondary prevention ICD indication, low LVEF, monomorphic VT as the triggering arrhythmia, and class I AAD use were all associated with ES occurrence. Of note, ICD implantation for secondary prevention was specifically associated with a three-fold increased risk of ES. Interestingly, however, only a trend towards increased prevalence rates of advanced age and male sex were observed, and no significant differences have been found between ischemic and non-ischemic etiologies.^7^ Other factors such as severely depressed LVEF and chronic kidney disease are also significantly correlated with ES development **(Table 1)**.^8^

### Initial evaluation and multidisciplinary approach

ES is a multidisciplinary syndrome requiring a multidisciplinary approach involving technical aspects such as ICD reprogramming, advanced intensive care in patients experiencing low-output state and multiorgan failure, interventional procedures such as radiofrequency (RF) CA and advanced HF management. Careful risk stratification and the early recognition of prognostic unfavorable signs are of pivotal importance in achieving success.

In patients who present with multiple clustered ICD shocks, the first step should always be to perform a device interrogation to exclude inappropriate shocks (ie, atrial fibrillation with fast ventricular response) and device reprogramming to reduce shocks in favor of ATP, which is painless. This can be reached both by increasing detection rate and duration.^9–11^

All potentially reversible causes of arrhythmias (ie, electrolyte imbalances, acute ischemia, proarrhythmic drug effects, hyperthyroidism, infections, and decompensated HF) should also be ruled out or treated.^12^ Finally, all patients should be risk-stratified according to hemodynamic tolerability of the arrhythmia and the presence of comorbidities.^13^ All patients with signs of hemodynamic decompensation (ie, persistent hypotension with the need for continuous infusion of vasopressors), as well as those patients with hemodynamically tolerated VT but who have major comorbidities such as LVEF ≤30%, moderate to severe chronic kidney disease, and/or severe obstructive pulmonary disease should be considered as high risk and admitted to the intensive care unit (ICU). The use of general anesthesia; mechanical ventilation; and/or circulatory support with an intra-aortic balloon pump (IABP), a left ventricular assist device (LVAD), or extracorporeal membrane oxygenation (ECMO) may be required in severe cases. Circulatory support with ECMO was recently shown to suppress ES in up to 62% of patients with refractory arrhythmias and cardiogenic shock and to prevent further secondary organ damage and maintain sufficient cardiac unloading.

**Figure 1:**
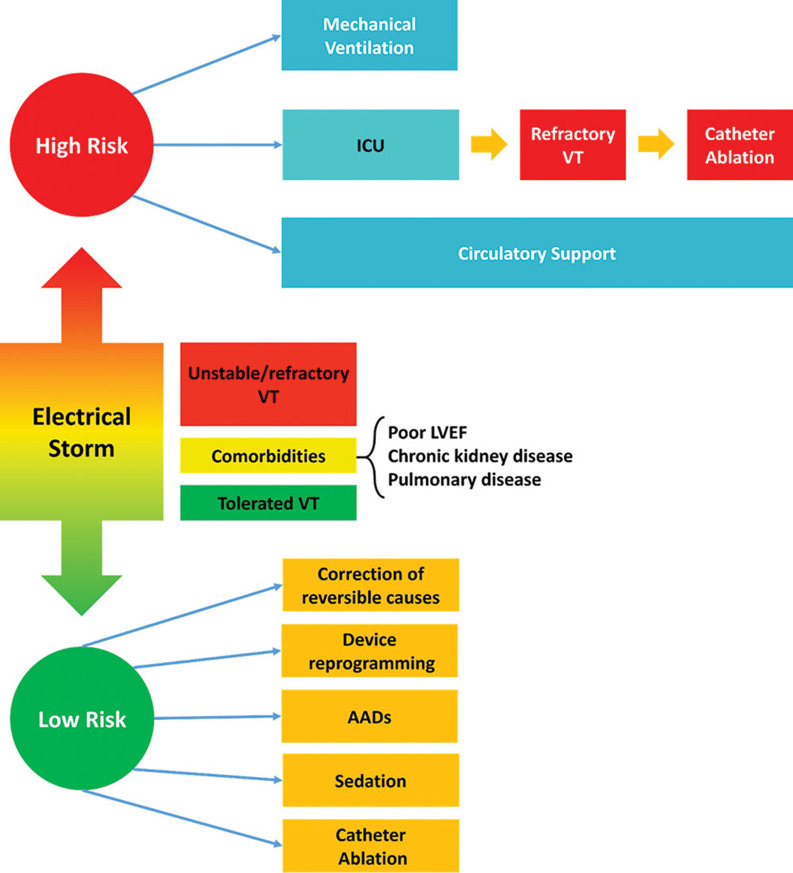
Risk stratification and management of patients presenting with ES.

Despite optimal management with hemodynamic support, the mortality rate of patients experiencing ES complicated by cardiogenic shock remains high (50%).^14^ In addition to VA suppression, hemodynamic support may allow for the use of multiple AADs (which may have negative inotropic effects) and facilitate the performance of invasive procedures including CA in hemodynamically compromised patients. A practical flowchart for initial patient care and risk stratification is presented in **Figure 1**.

### Pharmacologic therapy

The introduction of pharmacologic therapy with AADs can be effective in suppressing VAs and reducing the need for ICD-based therapies in patients with ES, even if a clear mortality benefit as compared with standard medical therapy has never been proved.^15^ The cornerstone of antiarrhythmic therapy is sympathicolysis with ß-blockers. A significant increase in sympathetic tone characterizes ES and is responsible for VA onset and maintenance, making the suppression of adrenergic tone pivotal.^16^ Most benefits of ß-blockers are related to their class effect. However, non-selective ß_1_ and ß_2_ blockage may have some advantages related to the higher penetration of some unselective ß-blockers like propranolol into the central nervous system, where they act by blocking presynaptic adrenergic receptors.^17,18^ Besides ß-blockers, sedation should be considered in all patients to minimize pain related to ICD shocks and reduce the sympathetic surge triggered by repeated ICD therapies. Benzodiazepines alone or in addition to short-acting analgesics such as remifentanil should be the first choice as they can suppress sympathetic hyperactivity and provide analgesia without negative inotropic effects.^19,20^ Propofol has been reported to suppress ES but must be used carefully since its negative inotropic effects can lead to cardiogenic shock.^21^ Dexmedetomidine is an α_2_-presynaptic receptor agonist that reduces sympathetic activity by enhancing central vagal tone and inhibiting presynaptic catecholamine release. However, it should be used cautiously since it can result in severe hypotension and bradycardia.^22,23^

Amiodarone is generally the AAD of choice for use in uncomplicated cardiac patients; it has been validated in numerous clinical trials and can be safely administered in the absence of contraindications like hyperthyroidism or QT prolongation. Thanks to its mixed antiarrhythmic class action (potassium, sodium, and L-calcium channels and sympathetic blocker), it can control VAs in up to 40% of patients undergoing intravenous administration, as well as reduce recurrent VT over follow-up.^24–27^ When administered intravenously, a central venous access method is strongly recommended because thrombophlebitis of peripheral veins is a well-recognized complication of intravenous amiodarone use in high doses (ie, 300 to 1,200 mg). Rarely, thrombophlebitis can result in systemic infection including bacteremia and device infections, which further complicate the clinical course in patients with ES.^28,29^ The combined use of amiodarone and ß-blockers significantly reduces the risk of recurrent ICD shocks compared with ß-blockers alone.^27^ Notably, amiodarone may increase the defibrillation threshold; therefore, ICD testing should be considered.^30^ Unfortunately, long-term amiodarone administration can cause potential side effects including liver dysfunction, thyroid disorders, pulmonary fibrosis, corneal deposits, and optic neuropathy.^31^ A recent pooled analysis of randomized controlled trials comparing CA and AAD performance revealed an association between amiodarone and increased mortality.^15^ Furthermore, we recently demonstrated that higher amiodarone dose at discharge following CA for VT in the setting of structural heart disease was associated with increased patient mortality, suggesting that discontinuation or dose reduction should always be pursued following successful CA.^32^ In the case of amiodarone failure, other drugs may be considered, such as procainamide, lidocaine, mexiletine, and sotalol. Procainamide is a class IC agent that may be helpful to acutely terminate VAs. Recently, in the PROCAMIO trial, the intravenous administration of procainamide, in comparison with amiodarone, was as safe and more effective in terminating tolerated monomorphic VT.^33,34^ The use of lidocaine in cases of ES remains limited due to its low efficacy in terminating scar-related VTs. During ischemic VT, the altered membrane potential and pH reduction increase the drug-binding rate, so lidocaine is mostly recommended for VA suppression in the setting of acute ischemia.^35,36^ Sotalol has been shown to reduce ICD shocks among patients who are implanted with ICDs for secondary prevention but has not been demonstrated to be superior to ß-blocker therapy according to several randomized controlled trials.^27,37,38^

### CA

The role of CA in VT management is becoming increasingly relevant, having repeatedly shown its superiority to medical therapy in reducing the arrhythmic burden and thus improving the prognosis and quality of life for patients with structural heart disease who present with VT.^15,39,40^ In the specific setting of ES, CA is effective both in the acute suppression of VAs and the long-term prevention of VT and ES recurrences **(Table 2)**.^41,42^ In a pooled meta-analysis of 471 patients with ES who were treated invasively, the acute elimination of all inducible VAs was achieved in 72% of cases, with clinical arrhythmias effectively suppressed in 91%. After a median follow-up of 1.2 years, 94% of patients were free from ES, and 72% experienced no further VA recurrences. In the VANISH trial, a trend towards a 34% relative risk reduction of ES recurrences was observed among 132 patients treated by CA, in comparison with 127 patients who were conservatively managed.^39^

We recently reported on the long-term outcomes of a large series of 267 patients presenting with ES undergoing CA. The acute elimination of all inducible VTs was achieved in 73% of the cases, with a 54% VT-free survival and a 93% ES-free survival rate at five years’ follow-up. Often, more than one CA procedure is needed to achieve good long-term VT control, especially in patients presenting with ES. In our experience, an average of 1.5 CA procedures per patient was necessary to achieve a 60-month VT-free survival period in more than half of the patients operated on.^43^ A recent multicenter analysis evaluating the safety and outcomes of repeated VT CA revealed that patients presenting with ES more often underwent multiple procedures (about 38% of the cases). Repeated procedures were generally longer, had more inducible and less mappable VTs, involved more epicardial access, and had higher complication rates (8.4% versus 4.8%) than initial procedures, with most complications related to pericardial or vascular access (approximately 2%).^44^ These data highlight the importance of vascular access management, particularly when the patient may need to undergo multiple procedures during the same hospitalization period. Femoral artery hemostasis can be achieved with either manual compression or vascular closure devices, with recent studies suggesting improved outcomes with the use of active closure systems.^45^ Even in patients experiencing VT recurrence, CA still substantially reduced the VT burden in most cases, with a median of 32 (15-55) VT episodes occurring during the six months leading up to the procedure, versus a median of 0 (0-1) VT episodes occurring in the six months after the procedure.^43^ Most available data come from patients who experience ES in the setting of ischemic cardiomyopathy and, less frequently, non-ischemic cardiomyopathy, with only limited information concerning specific situations like arrhythmogenic right ventricular cardiomyopathy (ARVC). The prevalence, therapeutic options, and appropriate implications of ES in ARVC patients are still not completely understood. Retrospective registries have shown that up to 30% of ARVC patients may suffer at least one episode of ES during their lifetime.^46^ In this setting, CA is considered the therapeutic option of choice, able to achieve a long-term VT-free survival rate of up to 80%.^40^ Since these patients often have severe right ventricle (RV) dysfunction, the use of left ventricle-only support devices such as IABP or percutaneous LVAD may be inadequate. Thus, when hemodynamic support is required in these individuals, the use of an RV support device or ECMO system can be considered.

**Figure 2:**
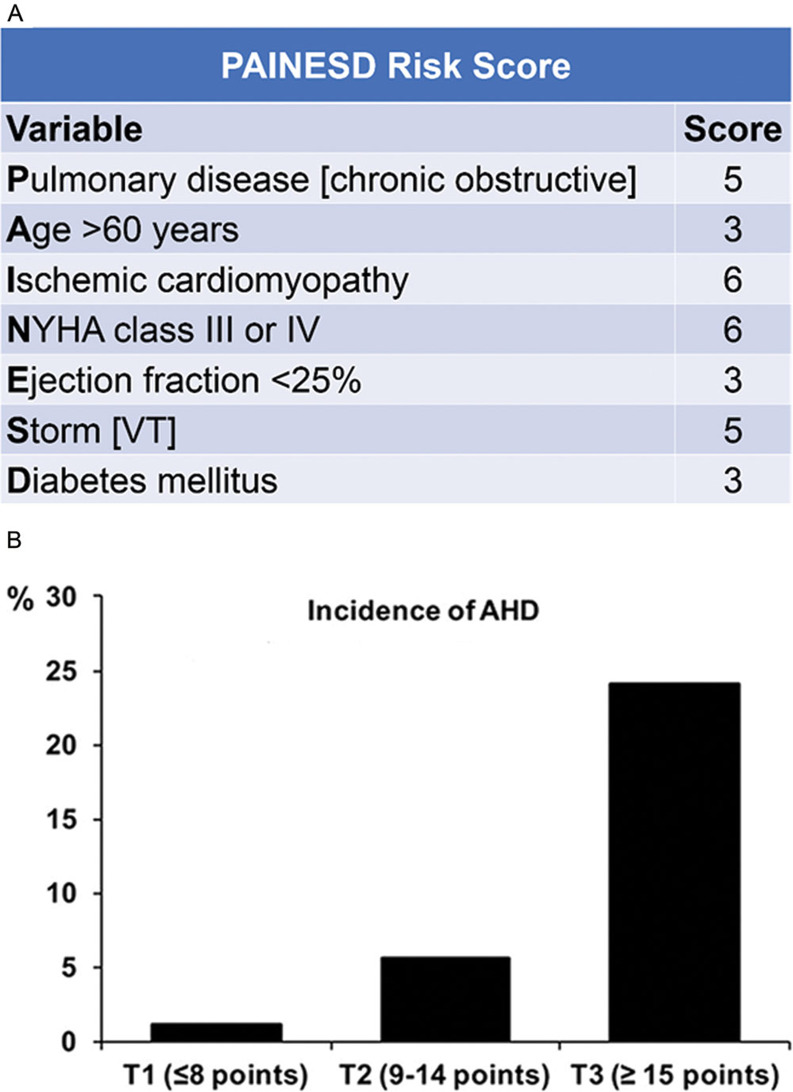
**A and B:** A scoring system to identify patients undergoing CA at high risk of hemodynamic decompensation who may benefit from prophylactic mechanical circulatory support, as proposed by Santangeli et al.^47^

Performing RF CA in patients with ES is challenging, as advanced HF, unstable VTs, and non-cardiac comorbidities may all contribute to a low-output state, increasing the risk of intraprocedural hemodynamic collapse and, consequently, periprocedural mortality.^47,48^ We have described how pulmonary chronic obstructive disease (COPD), age, ischemic cardiomyopathy, New York Heart Association class, LVEF, ES at presentation, and the presence of diabetes are all factors that relate to acute hemodynamic decompensation during VT ablation. Moreover, we proposed a scoring system (the PAINESD score) that accounts for such characteristics to identify high-risk patients in whom prophylactic mechanical support may improve outcomes **(Figure 2)**.^49^ The PAINESD score has been validated in a cohort of 93 patients who presented with VT in the setting of structural heart disease and who were divided into three groups—prophylactic, rescue, and no percutaneous LVAD placement—for evaluation.^50^ In this study, patients who underwent rescue LVAD had a significantly higher 30-day mortality rate (58%) compared with patients who underwent prophylactic LVAD (4%), even if they had similar PAINESD scores (mean: 17.8 versus 16.5). Moreover, 30-day mortality among patients who underwent prophylactic LVAD was superimposable to mortality among patients who were ablated without LVAD support (3%), even if the latter had a significantly lower PAINESD score (mean: 13.4), thus highlighting the importance of using prophylactic mechanical support in high-risk patients to improve postprocedural mortality.^50^ However, some patients with advanced HF have significant biventricular dysfunction, and LVAD support may be inadequate. In these cases, devices providing biventricular support, including ECMO, should be considered. In a recent study involving 64 patients undergoing CA of unstable VTs, the prophylactic use of ECMO allowed for procedure completion in 92% of patients, the achievement of VT non-inducibility in 69%, and an 88% overall survival rate at 21 months’ follow-up.^50^

### Sympathetic denervation

Patients with ES refractory to standard medical treatment and CA may benefit from bailout treatments like epidural anesthesia or cardiac sympathetic denervation (CSD). As stated above, sympathetic hyperactivity plays a critical role in VA onset and maintenance. Arrhythmia suppression may therefore be achieved by modulating neuraxial efferents to the heart.^51,52^ Sympathetic denervation was recently applied to control ES in patients with structural heart disease.^51,52^ The procedure is usually performed on the left side using a video-assisted thorascopic approach and entails the removal of the lower one-third of the stellate ganglion and T2 to T4 thoracic ganglia, leading to effective control of the arrhythmic burden in up to 56% of patients.^52^ Bilateral CSD was initially used in cases of failure of the left CSD. In a small study that involved six patients who underwent bilateral CSD following failed medical therapy, CA, and epidural anesthesia, at least a partial response was observed in 84% of the cases.^52^ In another recent series of 41 patients with refractory VT undergoing either left (n = 14) or bilateral (n = 27) CSD, a significant reduction of ICD shocks during a mean follow-up period of one year was observed in 90% of patients, with a significantly higher ICD shock-free survival rate of 48% noted in the bilateral CSD group compared with 30% in the left SCD group.^53^ In a recent multicenter registry that included 121 patients with structural heart disease who underwent left or bilateral CSD for refractory VT or ES, bilateral CSD was associated with a two-fold risk reduction of the combined event of sustained VT/ICD shock recurrence, death, and/or heart transplant as compared with in patients who underwent a left side-only procedure.^54^

### Alternative interventional approaches

In patients in whom RF CA has failed or proves challenging (eg, in the presence of mitral and/or aortic mechanical valves), alternative approaches like transcoronary ethanol ablation and surgical cryoablation have been described.^55,56^ We reported a 73% VT-free survival at one-year follow-up in a series of 20 consecutive patients with non-ischemic cardiomyopathy and VT refractory to conventional therapy who underwent surgical cryoablation.^55,56^ Transcoronary ethanol ablation performed through selective coronary angiography to identify the branches supplying the putative VT site of origin was recently reported in a series of 46 patients with VT related to structural heart disease and refractory to conventional CA.^55^ At least partial procedural success was reached in 66% of patients with 74% and 82% VT recurrence rates at 6- and 12-months follow-up, respectively.

### Palliative care

Widespread ICD use has considerably improved the life expectancy of patients with structural heart disease at risk of sudden death, but it has also created new challenges for affected patients, those individuals who are close to them, and for involved healthcare professionals, particularly when ICD carriers approach the end of their life. This may be due to progressive worsening of the underlying heart disease (ie, advanced heart failure) that cannot be improved by additional treatment or the development of another terminal condition such as cancer. In this setting, ICD operation may be more of a burden than a benefit.^57–59^ When available, a multidisciplinary end-of-life care service involving cardiologists, palliative care specialists, spiritual advisers, and social workers should always be consulted to create an environment that supports patients and those close to them in various aspects of end-of-life care. A clinical protocol should be developed to create a care plan to identify the patient’s views about device deactivation and advanced intensive care. Relatives should always be involved (with respect to confidentiality) to provide support to the patient and help discuss care goals. Several opportunities to discuss ICD deactivation and the ability to contribute meaningfully to a shared decision should be given to the patient because this topic is sensitive and difficult. The relative risks and benefits of continued ICD therapies should be continually reviewed, and ongoing discussions should be had with all critically ill patients.^59^

## Conclusions

ES is a life-threatening condition that requires a multimodal approach including optimal ICD reprogramming, pharmacologic therapy, interventional approaches aimed at modifying the arrhythmic substrate such as the use of RF CA, or techniques to suppress the sympathetic trigger such as cardiac sympathetic denervation. Currently, RF CA appears to be the most valuable strategy to acutely suppress arrhythmias and improve long-term arrhythmia-free survival; therefore, it should be considered in all patients presenting with ES, reserving alternative approaches like surgical cryoablation or transcoronary ethanol ablation to selected cases refractory to or unsuitable for CA.

## Figures and Tables

**Table 1:** Reversible Causes and Clinical Predictors of ES in ICD Patients

Potential Reversible Arrhythmic Triggers	Recognized Clinical Predictors of ES
Acute myocardial ischemia	Severely depressed LVEF
Electrolyte imbalances	Secondary prevention ICD indication
Decompensated HF	Use of class I AADs
Hyperthyroidism	Monomorphic VT as triggering arrhythmia
Infection and/or fever	Chronic kidney disease
Proarrhythmic drug effects	
Early postoperative period	

ES: electrical storm; ICD: implantable cardioverter-defibrillator; HF: heart failure; LVEF: left ventricular ejection fraction; AAD: antiarrhythmic drug; VT: ventricular tachycardia.

**Table 2:** Studies Analyzing the Role of CA in ES

Study	Number of Patients	EF	Acute Suppression of ES	VT Recurrence	ES Recurrence	Follow-up (in Months)
Carbicicchio et al. 2008^41^	95	36% ± 11%	89%	34%	8%	Median: 22
Di Biase et al. 2012^42^	92	27% ± 5%	100%	34%	0%	25 ± 10
Muser et al. 2017^43^	267	29% ± 13%	73%	33%	5%	Median: 45
Sra et al. 2001^60^	19	27% ± 8%	87%	37%	–	7 ± 2
Silva et al. 2004^61^	14	31% ± 13%	80%	13%	–	12 ± 17
Arya et al. 2010^62^	13	33% ± 9%	100%	38%	–	Median: 23
Pluta et al. 2010^63^	21	–	81%	19%	0%	3
Deneke et al. 2011^64^	31	28% ± 15%	94%	25%	12%	Median: 15
Kozeluhova et al. 2011^65^	50	29% ± 11%	85%	52%	26%	18 ± 16
Kozluk et al. 2011^66^	24	27% ± 7%	–	34%	12%	28 ± 16
Izquierdo et al. 2012^67^	23	34% ± 10%	56%	–	35%	Median: 18
Jin et al 2015^68^	40	21% ± 7%	80%	53%	–	17 ± 17
Kumar et al. 2017^69^	287	ICM: 27% ± 10% NICM: 33% ± 16%	ICM: 60% NICM: 50%	ICM: 49% NICM: 64%	ICM: 17% NICM: 27%	Median: 42

CA: catheter ablation; EF: ejection fraction; ES: electrical storm; ICM: ischemic cardiomyopathy; NICM: non-ischemic cardiomyopathy; VT: ventricular tachycardia.
